# Comparing Constraints on Contraction Using Bayesian Regression Modeling

**DOI:** 10.3389/frai.2020.00058

**Published:** 2020-08-12

**Authors:** Laurel MacKenzie

**Affiliations:** Department of Linguistics, New York University, New York, NY, United States

**Keywords:** contraction, English, copula, linguistic variable, Bayesian modeling, multinomial regression

## Abstract

This paper has three goals: (1) to document the factors shaping *is*-contraction in Mainstream American English; (2) to assess the extent to which these factors also shape contraction of *has*; (3) to use shared patterns of contraction across the two verbs to draw conclusions about how the varying forms are represented grammatically. While *is* has two distinct phonological forms in variation, *has* has three. This necessitates regression modeling which can handle non-binary response variables; I use Bayesian Markov chain Monte Carlo modeling. Through this modeling, I (1) uncover a number of novel predictors shaping contraction of *is*, and (2) demonstrate that many of the patterns shown by *is* are also in evidence for *has*. I also (3) argue that modeling *has*-variation as the product of two stages of binary choices—a common treatment of three-way variation in variationist sociolinguistics—cannot adequately explain the quantitative patterns, which are only compatible with a grammatical model under which three distinct forms vary with each other. The findings have theoretical and methodological consequences for sociolinguistic work on ternary variables.

## 1. Introduction

Several English verbs can surface in at least two forms: one with all phonological material intact (e.g., [ɪz]), and one which is phonologically reduced and cliticized to its host (e.g., [z]). The variation between these two forms, known as contraction, has been investigated in a wide variety of corpora of both spoken and written language. This research has primarily focused on identifying the factors which condition one case of contraction in particular: the contraction of tensed forms of *be*, predominantly *is*.

Early work sought to identify the relative contributions of phonological, syntactic, and sociostylistic factors in the contraction of tensed *be*; later work has brought usage-based factors, such as predictability and persistence, into the picture. One particularly influential strand of work has compared the patterns of tensed *be* contraction in Mainstream American English to the patterns shown by tensed *be* absence in African American Vernacular English, and used the parallels between them to conclude that comparable processes drive the variation in both varieties. Studies of the constraints on contraction have shed light on broad theoretical questions about the nature of grammar; these include whether and how morphosyntax and phonology can interact (Anttila, 2016), and the extent to which grammar incorporates usage-based and processing constraints (Spencer, 2014).

Contraction of tensed verbs other than *be* has been less often examined. However, comparing patterns of contraction across different verbs can fill in our picture of how English verbs vary, and can answer questions about the generalizability of certain patterns which have thus far been attested only for *be*. This is turn speaks to questions of how variable phenomena are represented in the grammar, questions which have been addressed using contraction data since Labov (1969).

This paper contributes to developing a comprehensive understanding of tensed verb contraction in Mainstream American English (MAE) beyond the patterns evinced by tensed *be* alone. First, I pull together a variety of findings, not all of which have yet been considered together, on the factors that condition *is*-contraction. I examine their patterning on the largest data set of post-noun phrase *is*-contraction in spoken MAE to date.

Then, I explore the parallels between contraction of *is* and another verb which contracts in a similar way: *has*. Both verbs can surface in a non-syllabic form, represented in orthography as '*s*: an alveolar sibilant which agrees in voicing with the preceding segment. But *has* differs from *is* in a crucial way: when produced in spoken MAE, *has* has **two** possible syllabic realizations, one with an onset ([həz]) and one without ([əz]). Ternary variation like this complicates both variationist theory—where sociolinguistic variables are commonly represented as binary choices, even when this requires conflating two surface forms and opposing them to the third—and variationist methods—with traditional logistic regression analysis accommodating no more than two possible outcomes. For this reason, no study to date has yet adequately analyzed the quantitative patterning of *has* in spoken language in a way that recognizes its three unique surface realizations **and** allows all three possible forms to vary independently. The present study does this, capitalizing on a recent movement employing Bayesian multinomial modeling for the analysis of ternary linguistic variation (e.g., Levshina, 2016; Grafmiller et al., 2018; Dilley et al., 2019).

Doing this allows me to address an additional issue: the underlying representation of the three different forms of *has*. In previous work (MacKenzie, 2013), I argued in favor of a treatment under which the tripartite variation in *has* was best understood as deriving from a cascade of two binary choices. The three forms on the surface mapped onto two forms underlyingly; the third form was derivative from one of the two selected in the first-stage choice. This, I argued, explained certain quantitative patterns in the data. When this analysis was assumed, the rate at which different forms of *has* were used in different contexts paralleled the rates for the analogous forms of *is*, suggesting unity of the two contraction processes at an abstract level. That analysis, though, considered only two contextual effects on the contraction of *is* and *has*. The present study considers thirteen. This not only expands the testing ground on which to look for analogous behavior in the patterning of the two verbs; it deepens our understanding of the contextual factors that affect variation in these two verbs in the first place.

In this paper, I answer the following questions:

1. Which factors condition the alternation between contracted and full forms of *is*?
2. Do those same factors condition the alternation between the contracted form of *has* and its two other possible forms?
3. Does the patterning of *has* lend support to the analysis under which the three surface forms are derived from two stages of binary choice?

These questions echo Labov's (1969, p. 760) research program of identifying “the most general form of linguistic rule” when similar-seeming patterns recur across different variable phenomena. Shared patterns of variation can be taken as evidence for structural unity of varying items in speakers' mental grammars. In fact, I do find that there are a number of shared patterns between contraction of *is* and contraction of *has*. But I also find that the *has* patterns cannot be fully accommodated under the earlier two-stage analysis. The forms of *has* appear to vary in a ternary way, complicating our understanding of what a linguistic variable can look like.

The findings have relevance not only for studies of tensed verb contraction in English, but for longstanding questions of the nature and representation of variable phenomena. They additionally carry methodological importance for variationist sociolinguistics. It is not uncommon for researchers, when faced with an alternation that is more than binary, to group variants together for regression analysis. However, a longstanding theoretical tenet of variationist research is that regression models are meant to represent grammar (Cedergren and Sankoff, 1974). Grouping two variants together and opposing them to a third implies that, at some level, the speaker makes such a choice. Grouping as a methodological exigency thus has theoretical consequences that may be unwarranted. In the present paper, I demonstrate that allowing three variants to vary independently in a statistical model can shed light on the relationship between those variants without the researcher having to impose any such relationship on the analysis. Accordingly, the paper includes a call to action for variationist sociolinguists who work with non-binary variables to branch out into different modeling techniques.

## 2. Background

### 2.1. Analyzing Contraction

The variation under study in this paper is the phonological realization of two tensed English verbs: *is* and *has*. These verbs can variably surface in a number of phonologically distinct forms. The verb *is* has two distinct forms in which it can surface: a single-consonant form (which agrees in voicing with the preceding segment), and a syllabic form, as shown in 2^1^. The following examples are taken from the Switchboard Corpus (Godfrey and Holliman, 1997; see section 3.1 for more on the corpus).

(1) Forms of *is*.
  Yeah, Salzburg'[z] nice. Austria'[z] nice. Europe [əz] nice!
  (sw1151)^2^
  [z]: single-consonant form
  [əz]: syllabic form

The verb *has* has three distinct forms in which it can surface: a single-consonant form, a syllabic form with no onset, and a syllabic form with an onset, as shown in 2.

(2) Forms of *has*.
  This spring [həz] been a little hard to keep up the pace because we've had a lot of family activities: my wife [əz] taken up skiing […] she[z] taken up snow skiing.
  (sw1402)
  [z]: single-consonant form
  [əz]: syllabic form, no onset [həz]: syllabic form with onset

In other words, *is* and *has* both show variation between a single-consonant form ([z]) and a form with all phonological material intact (for *is*, [əz]; for *has*, [həz]). But *has* differs from *is* in additionally allowing a third variant, the syllabic, onsetless [əz].

For ease of exposition, I give the three different forms unique names (Table 1). I follow the literature in using the term “contraction” to refer to the alternation between the contracted form and any other form(s) of a given verb.

**Table 1:** Forms of *is* and *has*.

**Name**	**Description**	**Example**
Contracted form	Single-consonant form	[z] “is,” “has”
Full form	Phonologically intact form	[əz] “is,” [həz] “has”
Intermediate form	Third form (*has* only)	[əz] “has”

In previous work (MacKenzie, 2013), I followed a sizable body of work in phonology and morphology and analyzed *is*-contraction as variable allomorphy. In other words, when producing the third singular present form of *be*, a speaker has a choice between two options: one that consists of a single consonant (the contracted form), and one with all phonological material intact (the full form).

This then raises the question of what kind of choice a speaker has when going to produce the third singular present form of auxiliary *have*. MacKenzie (2013) considers two possibilities. The first, a ternary analysis, treats all three surface forms of *has* as represented underlyingly. Variation in *has* realization under this approach would consist of variable three-way allomorphy: a choice between full, contracted, and intermediate forms. The second possibility consists of two binary choices: first, a choice between the contracted form and the full form, as for *is*; then, in cases where the full form has been selected, a second choice between producing the full form as-is or reducing it to the intermediate form via /h/-deletion, an independently attested fast-speech reduction phenomenon in English. This second possibility brings contraction of *has* more in line with contraction of *is*. It also suggests that the choice between full and contracted forms, which takes place in a similar way underlyingly for the two verbs, may pattern in the same way on the surface. Indeed, this is what I found in MacKenzie (2013). Contracted forms of *is* and *has* are selected at very similar rates for the two verbs. Additionally, contracted forms of *is* and *has* both show identical effects of being dispreferred after longer noun phrase hosts. By contrast, the choice between intermediate and full forms of *has* shows no sensitivity to host phrase length. This suggests that the factor of host phrase length operates on a choice between contracted and full, and not on a later choice point that may occur between full and intermediate.

Still unresolved is whether the other factors that condition contracted forms of *is*—which will be detailed in the following section and confirmed in the first set of results presented in this paper—operate in the same way on contracted, but not intermediate or full, forms of *has*. If they do, this will constitute even more evidence in favor of the two-stage analysis of *has* presented in MacKenzie (2013).

To set the stage for this analysis, I survey the existing work on contraction of *is* in the next subsection.

### 2.2. Variation in Tensed *Be*

The bulk of quantitative corpus research on contraction addresses variation in tensed *be*, and within that, there is considerable research on contraction of the third singular form *is*. Despite some differences in the data used across different studies—spoken vs. written language, sociolinguistic interviews vs. telephone conversations—several key factors are consistently found to shape the alternation between *is* and '*s*. Many of these have to do with the nature of what I will call the “host phrase” of the contractable verb: the phrase onto which the verb cliticizes when it contracts^3^. Also relevant are properties of what I call the “host word”—the word immediately preceding the contractable verb—and the verb's complement—the constituent following the contractable verb.

One of the strongest effects on contraction of *is* is whether the verb's host phrase is a pronoun or a non-pronominal noun phrase (henceforth “NP”). Speakers use the contracted form of *is* at near-categorical rates after pronominal host phrases, and much lower rates after NP host phrases (Labov, 1969; Rickford et al., 1991; McElhinny, 1993; MacKenzie, 2013; Barth and Kapatsinski, 2014; Spencer, 2014; Bresnan, 2018). Due to this near-invariance, some researchers have opted to analyze *is*-contraction after NP host phrases separately from pronominal host phrases, or even dispense with post-pronominal data altogether, because the contraction rate is so high. I adopt the latter approach in the present study, examining contraction only after NP host phrases. In section 3.2, I describe the exclusion criteria I used to achieve this.

Another strong effect on *is*-contraction is the length of an NP host phrase. Even with pronominal host phrases excluded, longer host phrases disfavor contraction (MacKenzie, 2013; Spencer, 2014; Bresnan, 2018). Host phrase length can be operationalized in a number of different ways; there is some evidence that orthographic word count predicts the variation somewhat better than other measures (MacKenzie, 2012, chapter 5).

Semantic and phonological characteristics of the host phrase also play a role in conditioning the variation. *Is*-contraction has been found to be sensitive to host phrase animacy, with more contraction after human-referent than non-human referent host phrases, an effect that is not reducible to a confound with proper noun status (McLaughlin, 2014). And studies generally find *is* to contract more after an immediately-preceding vowel than a consonant, and more after a voiced consonant than a voiceless one (Labov, 1969; MacKenzie, 2012; Spencer, 2014). Though surrounding syllable stress has been hypothesized to play a role in contraction (Anttila, 2017), it has not been found to affect *is*-contraction in the two studies that have looked (MacKenzie, 2012; Bresnan, 2018).

Additionally, a widely-discussed effect on *is*-contraction is the syntax of the verb's complement. The sociolinguistic literature on *is*-variation in African American Vernacular English (AAVE)—which allows both *is*-contraction and *is*-deletion—has tended to differentiate five complement types: nominal, locative, adjectival, progressive verb, and future *gonna/going to*. Deletion of *is* in AAVE shows clear sensitivity to this factor, with the ordering of environments given in the previous sentence reflecting a commonly replicated hierarchy from least deletion-favoring to most deletion-favoring (Sharma and Rickford, 2009). However, contraction of *is*, in both AAVE and MAE, has shown less clear patterning, and it is difficult to compare across studies which have operationalized this factor in different ways. That said, there is a general trend by which verbal complements—progressive verbs and/or futures—favor contraction more than other complements (Labov, 1969; McElhinny, 1993; Barth and Kapatsinski, 2014; Spencer, 2014; MacKenzie, 2016).

Well-studied in recent literature are measures of the predictability, or conditional probability, of the contractable verb given surrounding words. Some researchers have found that *is*-contraction is more likely when the verb is highly probable given one or two surrounding words (Frank and Jaeger, 2008; Barth and Kapatsinski, 2014; Spencer, 2014), though the results depend on whether pronominal subjects are included in the analysis or not. There is also evidence that *is*-contraction displays persistence, that is, that speakers show a tendency to reuse whichever variant of *is* was previously used (Barth and Kapatsinski, 2014; Spencer, 2014; Bresnan, 2018). Though a few studies have considered the effect of speaking rate on *is*-contraction (Frank and Jaeger, 2008; MacKenzie, 2012; Spencer, 2014), it does not show a convincing, statistically significant effect in any of them.

Finally, where sociodemographic factors are concerned, there is some evidence that *is*-contraction shows effects of speaker sex/gender—with speakers identified by the corpus as male using contraction more than those identified as female (MacKenzie, 2012)—and speaker age, with younger speakers contracting more than older ones (Rickford et al., 1991; MacKenzie, 2012; Bresnan, 2018). At the same time, there is no evidence that speakers style-shift *is*-contraction in speech (Finegan and Biber, 1986; McElhinny, 1993; MacKenzie, 2012).

### 2.3. Variation in Other Tensed Verbs

There is much less research on the variable phonological realization of other tensed verbs, including auxiliary *has*, the other verb analyzed in this paper. Where *has* has been examined, researchers have generally opposed the single-consonant “contracted” form ([z] or [s] depending on voicing of the preceding segment) to the other forms (“intermediate” [əz], “full” [həz]) (McElhinny, 1993; Frank and Jaeger, 2008). This seems to presume a particular analysis of variant choice—that speakers make a binary choice between the contracted form and the other two combined—though this is not made explicit. The results are also difficult to generalize over, due to small token counts (McElhinny, 1993 examines only 76 tokens of *has*) and to researchers collapsing across forms (Frank and Jaeger, 2008 analyze *has, have*, and *had* together). Nevertheless, we can glean some patterns. *Has*-contraction shows the same favoring effect of a pronoun (as opposed to an NP) host phrase as *is*-contraction, and, among NP host phrases, the same effect of host phrase length in words (McElhinny, 1993; Frank and Jaeger, 2008; MacKenzie, 2012). Frank and Jaeger additionally find an effect of verb predictability, in keeping with that found for *is*-contraction. Analyses of preceding segment, speaking rate, and speaker sociodemographic factors are inconclusive, with some of the aforementioned three studies finding them, and others not.

### 2.4. Current Contribution

As the previous subsection emphasized, the present paper is virtually unique in analyzing contraction of *has* alongside the very similar contraction of *is*. Research that has compared these two verbs has not operationalized the forms of *has* as I do here, i.e., as three distinct forms that may vary independently.

In addition, the present paper expands our body of knowledge on the contraction of *is*. Though much research on *is*-contraction exists, the present study improves upon previous studies in two key ways. First, the present paper uses auditory coding of the variation, rather than relying on transcripts, which may not accurately reflect spoken language. Second, compared to other studies of *is*-contraction that do make use of auditorily-coded data, the present paper employs a much larger data base. Even though the data has been restricted to only those tokens of *is* with NP host phrases, my data base of 5,642 tokens is four times as large as that of Bresnan (2018), and nearly 10 times as large as that of Spencer (2014). This allows for increased statistical power, and uncovers novel results.

Finally, I see the present paper as making important methodological and theoretical contributions where the study of non-binary variation is concerned. Variables with more than two variants have long posed a problem for sociolinguistic research, for reasons of method—logistic regression models require outcome variables to be binary—and for reasons of theory—the original conception of the variable rule was that a single input variably yielded a single output (Cedergren and Sankoff, 1974; Wolfram, 1991). To get around these problems, researchers have resorted to strategically grouping variants together. So, in cases of ternary variation, researchers will combine data from two variants and oppose them to the third: see, for instance, a large literature on /t/ variation in regional British Englishes, where attested forms of /t/ include [t], [tʔ], and [ʔ], and various grouping strategies are employed (e.g., Foulkes et al., 2005; Straw and Patrick, 2007; Drummond, 2011). But it is not often acknowledged that grouping variants carries implicit theoretical assumptions about the structure of variation. A longstanding theoretical tenet of variationist research is that regression models are meant to represent grammar (Cedergren and Sankoff, 1974). While the earliest work to group non-binary variables explicitly linked the grouping procedure to a particular theoretical treatment of the variation (Labov, 1969), many more recent studies that group don't recognize the tacit grammatical claims that their grouping implies.

Another problem with grouping is that it can present a misleading picture of the constraints on variation. This was notably pointed out by Rickford et al. (1991) in their critique of Labov's (1969) study of copula contraction and deletion in AAVE. Labov defined copula “contraction” by opposing contracted and deleted forms of the copula to full forms, because, the theory went, all deletions had contracted forms at some point in their derivational history. He defined copula “deletion” by opposing deleted to contracted forms, omitting full. He then demonstrated that contraction and deletion were conditioned in the same way, which he argued supported an analysis under which contracted and deleted forms shared an underlying representation, and hence justified his grouping. But, as Rickford et al. pointed out, when contraction is calculated by grouping together contracted and deleted forms, it will inevitably be influenced by the patterns of deletion, particularly in cases where deleted forms greatly outnumber contracted ones.

I suggest that, when faced with non-binary variation like this, multinomial regression modeling is an important alternative to grouping, both in cases where a researcher does not want to impose a particular theoretical analysis on the data (such as AAVE copula variation, see McLaughlin, 2014), and in cases where there is no immediately obvious two-stage analysis to be imposed (such as English ternary genitive variation, see Szmrecsanyi et al., 2016). As an additional point in its favor, multinomial regression modeling has been found to explain variation as well as models that assume two stages of binary choice, at least for some variables (Sankoff and Rousseau, 1989).

In this paper, I take the multinomial model of *has*, under which all three forms are allowed to vary independently, as a null hypothesis. Then, I compare the factors that condition speakers' choices between contracted and the other two forms of *has* to the factors that condition speakers' choice between contracted and full forms of *is*. If contracted forms of *has* behave in opposition to the other two forms, and they are conditioned in similar ways to contracted forms of *is*, we have evidence to support the analysis of *has* variation under which speakers make a first-stage choice between contracted and full—just as they do for *is*—and then, where applicable, a second-stage choice between full and intermediate.

## 3. Methods

### 3.1. The Data

The data for the present study come from the Switchboard Corpus (Godfrey and Holliman, 1997). Switchboard is a transcribed corpus of telephone conversations between 542 native speakers of American English, paired at random by a robotic operator and assigned a topic to elicit a 5- to 10-min conversation. The corpus was collected between 1991 and 1992, and consists of about 240 h of speech (roughly 3 million transcribed words) across approximately 2,400 conversations.

Data were collected as described in section 3.2, and coded for the predictors enumerated in section 3.3. Data points with an NA value for any of the predictors of interest were omitted from analysis. This resulted in 5,642 tokens of *is* and 699 tokens of *has*.

### 3.2. Defining the Variable Context and Data Extraction

As mentioned in section 2.2, data for the present study were restricted to only those tokens of *is* and *has* following non-pronoun subjects. To this end, it was important to identify what counted as a pronoun. Data was excluded from the present study if the host phrase was any of the following: a personal pronoun (e.g., *she, he*), an expletive pronoun (*there, it*), a *wh*-pronoun (e.g., *what, who, where, whatever*), a demonstrative pronoun (e.g., *that*), an indefinite pronoun (e.g., *everybody, someone, anything, one*), a possessive pronoun (e.g., *mine*), or the locative *here*. This is a more conservative definition of what counts as a “pronoun host phrase” than others have used, but it is justified by the finding that pronoun-like host phrases, such as indefinite pronouns, have significantly higher rates of contraction than single-word NP hosts (MacKenzie, 2012). This suggests that contraction shows special behavior when the host phrase is a closed-class lexical item; for that reason, I omit any data points where the verb's host phrase is anything pronoun-like. Though this removes a relevant factor in the choice of contracted form, we are still left with a number of other factors to examine.

The first step in obtaining data was to search the corpus for the variants of each verb when occurring in non-post-pronominal contexts. This was done using a Python script. The script searched for tokens of the targeted verbs whose immediately preceding word did not fall into the category of pronouns listed above. To filter out tokens of main verb *has*, which does not contract in American English (Hughes et al., 2012, p. 23), the script returned hits for *has* and '*s* only when followed by a past participle with no more than three words optionally intervening. Past participles were defined as any word ending in *-en* or *-ed*, or on a list of 129 irregular past participles (e.g., *begun, gone*). All instances of *has* were scrutinized, and tokens of main verb *has* that slipped through were removed from the data.

To target forms of *is*, the script searched for *is* and '*s*. All instances of '*s*, which can reflect several different morphemes in English, were scrutinized. Instances of '*s* that were actually contracted forms of *has* were retained in the data only when they had not been picked up by the previous search. Instances of '*s* that were actually the possessive morpheme were removed from the data.

After this initial stage of data collection, the second step was to eliminate data points where *is* or *has* occurred in an environment where contraction is blocked. This follows traditional variationist methodology, and ensures that the analyst only studies those environments in which each form of the dependent variable is grammatical, preventing results from being skewed (Labov, 1972; Tagliamonte, 2006). The numerous environments where the full range of variants of *is* and *has* is prevented from surfacing can be found in MacKenzie (2012), chapter 3, and references cited therein.

Tokens were also omitted from study when they contained a negated verb, since three variants are possible there (e.g., *is not*, '*s not*, or *isn't*). Finally, a single Switchboard speaker was observed to use copula deletion; tokens from this speaker were omitted, since the availability of this third variant of *is* skews the distribution of forms relative to other speakers.

Once the data had been obtained, the author listened to each instance of *is/has* in the data and coded each occurrence of the dependent variable as contracted, full, or (*has* only) intermediate. As part of this stage of data extraction, tokens were excluded if the verb was contrastively stressed or if the speaker paused between the host word and the verb (MacKenzie, 2012).

Even though contraction of *is* and *has* is represented orthographically in English, it was important to listen to each instance of the dependent variable and code it auditorily, rather than relying on Switchboard transcriptions. There were two reasons for this. First, there is no standardized way of representing the intermediate form of *has* ([əz]) orthographically, but it is still a phonologically distinct variant and should be coded as such. In fact, the vast majority of tokens identified by the author as phonologically intermediate were orthographically represented in the transcriptions as full forms (179 out of 182). Second, there is reason to believe that Switchboard's transcriptions of contracted and full forms are not reliable. According to the Switchboard manual, transcribers were “always permitted to spell out forms in full, even if the pronunciation suggests the contracted form.” Indeed, of the forms of *has* and *is* that were identified by the author as contracted, a sizable proportion of them were found to have been transcribed as full by Switchboard's transcribers (*has*: 21%; *is*: 35%). (Forms identified by the author as full were indeed transcribed as such, at a rate of 100% for *has* and 99% for *is*). For this reason, auditory coding of the dependent variable was essential. The author carried out all such coding.

Finally, each data point was coded by the author or a trained research assistant for a number of predictors, described in the following subsection.

### 3.3. Predictors

Modeling included three random intercepts: speaker, word preceding the target, word following the target. (Here and henceforth, “target” refers to the contractable verb.) Speakers and words with five or fewer observations in the data were recoded as “other” following Levshina (2016, p. 253). The fixed-effect predictors coded for were:

- **Host phrase length in orthographic words**: a continuous measure.
- **Host phrase humanness**: categorical, treatment coded, with three levels, following Rosenbach (2005) and Wolk et al. (2013): **human** (default); **collective**, comprising organizations and “temporally stable groups of humans with potentially variable concord” (Wolk et al., 2013, 396); **non-human**.
- **Host phrase proper nounhood**: categorical, treatment coded, with two levels, **no** (default) and **yes**.
- **Preceding segment**: categorical, sum coded, with levels **voiced consonant, voiceless consonant, high vowel, other vowel, R**. Segments were identified based on a transcription of the preceding word taken from the CMU Dictionary v.0.7 (Weide, 2008). Words not in the dictionary were transcribed by hand. The subdivision of consonants by voicing is informed by previous findings (e.g., Spencer, 2014). The subdivision of vowels into high and other was based on my experience coding the dependent variable: I often had difficulty determining whether an instance of *is* following a high vowel was contracted or not^4^. /ɹ/-colored final vowels were given their own category due to uncertainty concerning whether they should be considered vowels or consonants.
- **Preceding and following syllable stress**: categorical, sum coded, with levels **monosyllabic, primary, secondary, unstressed**. Syllable stress was obtained based on the transcriptions provided in the CMU Dictionary v.0.7. Words not in the dictionary were transcribed by hand. Due to small Ns, the *primary* and *secondary* categories of following syllable stress were combined as *stressed* for *has*.
- **Complement syntax (*is* only)**: categorical, sum coded, with levels **unknown** (speaker changes direction or restarts), **noun phrase** (including gerunds), **determiner phrase, quantifier phrase**^5^, ***wh*-phrase, past participle, adjective phrase, number phrase, prepositional phrase, locative prepositional phrase, progressive, future**. Cases where a disfluency and/or an adverb immediately followed the target were coded for the syntax of the constituent following the disfluency/adverb. This is a larger number of categories than has been identified in previous studies, but ambiguity in previous researchers' methods made it difficult to apply any previous coding scheme to the present data, so the decision was made to err on the side of caution and make more distinctions than were potentially necessary. Still, some issues remain with the coding: for instance, *about to* (as in *Summer's about to be here*) was coded as a prepositional phrase, but semantically, it has a future meaning. Ascertaining the behavior of such syntactically–semantically mismatching following constituents is an interesting direction for future work.
- **Speaker sex/gender**: categorical, sum coded, with levels **male, female** based on the information provided in the corpus.
- **Speaker year of birth**: continuous, centered around the median, rescaled to decades.
- **Previous form of verb**: categorical, treatment coded, with levels **none** (default), **full, contracted, intermediate** (for *has* only). This predictor checks for persistence, and compares the likelihood of contraction of tokens that follow a previous instance of the verb to the likelihood of contraction of the first token of a conversation. Coding for this predictor was done on a speaker-by-speaker basis, so cross-speaker persistence or accommodation effects were not allowed for. Instances of *has* where it was functioning as a main verb were not counted as previous forms of *has*. Also uncounted were instances of '*s* where it was functioning as a possessive marker or as a contracted form of the other verb (e.g., contracted *is* when the target was *has*, etc.). Future work can explore the possibility of persistence effects between phonologically identical but morphologically distinct forms like these (and see also Tamminga, 2016).
- **Relative speaking rate**: a continuous measure reflecting the ratio between the speaking rate of the annotation unit containing the target and the speaker's average speaking rate across the entire corpus. The higher the value, the faster the speech is, relative to the speaker's average.
- **Following disfluency**: categorical, treatment coded, with levels **no** (default) and **yes**, reflecting whether the word immediately following the target was *uh* or *um*.
- **Forward transitional probability**: a continuous measure reflecting the conditional probability of the target given the preceding word. Calculated as the corpus-internal frequency of the preceding word + target bigram divided by the frequency of the preceding word. Log-transformed.
- **Backward transitional probability:** a continuous measure reflecting the conditional probability of the target given the following word. Calculated as the corpus-internal frequency of the target + following word bigram divided by the frequency of the following word. Log-transformed.

Spearman correlations were used to check continuous predictors for collinearity. For both verbs, host phrase length and forward transitional probability were found to be weakly negatively correlated (rho = −0.328 for *is*, rho = −0.318 for *has*). This is unsurprising: longer subjects are more likely to be structurally complex, with embedded phrases causing them to end in items like verbs and prepositions, which are unlikely to be themselves followed by a(nother) verb. Accordingly, the log-transformed measure of forward transitional probability was residualized by host phrase length for each verb, and this residualized predictor was used in modeling.

### 3.4. Modeling

While *is*-contraction is easily modeled using the binomial (two-outcome) mixed-effects logistic regression models that have become commonplace in variationist research, the three-way variation shown by *has* is not. As Sankoff and Rousseau (1989, 6) observe, there are two fundamental approaches to modeling a three-variant variable like this: as a single choice between three options each time a speaker goes to produce a form, or as two binomial choices: first between one form and the other two combined, and then between those latter two forms. Like *is*-contraction, the second of these two options can be easily modeled with (two rounds of) binomial logistic regression, but at the downside of imposing a particular analysis on the data (see section 2.4).

For this reason, I analyze *has*-contraction with multinomial logistic regression, a simple extension of binomial logistic regression which allows the user to compare a reference or default category to each of the other possible outcomes (Levshina, 2015b). And, in order to accommodate the inclusion of random effects, which have been argued to be essential in sociolinguistic research (Johnson, 2009), I implement Bayesian modeling using R's MCMCglmm package (Hadfield, 2010a). For consistency, I use Bayesian modeling for both verbs: a binomial model for *is*, and a multinomial one for *has*. Recent linguistic papers that make use of multinomial MCMCglmms include Levshina (2016), Grafmiller et al. (2018), and Dilley et al. (2019). For a detailed description of the philosophy and methodology behind these models that is geared to a linguistic audience, the reader is pointed to the first two of these articles. Levshina (2015a) provides a brief tutorial, again for a linguistic audience, on getting started with MCMCglmm modeling; for a more detailed tutorial and primer on these models, consult Hadfield (2010a,b, 2019). Below, I briefly describe these models and summarize what distinguishes them from the logistic regression models that sociolinguists are used to.

MCMCglmm implements Bayesian Markov chain Monte Carlo modeling. The models used here are Bayesian in that they require the user to specify prior beliefs about the probability distributions of the model parameters; after considering the data, they output posterior probability distributions for each parameter of interest. The models also make use of Markov chain Monte Carlo methods to estimate the posterior probabilities, generating representative random values from these distributions and then approximating the posterior probability distributions from these values. The output of an MCMCglmm model can be interpreted like the output of a logistic regression model fit with lme4 in R (R Core Team, 2017): coefficients in log odds are provided for the different levels of each categorical independent variable; these indicate the change in log odds associated with that level of using the non-default variant of the dependent variable. For continuous predictors, coefficients reflect the change in log odds of using the non-default variant of the dependent variable with each one-unit increase of the predictor. Positive coefficients reflect a change in log odds in favor of the non-default variant; negative coefficients reflect a change in log odds in favor of the default variant, or reference level. However, unlike in traditional logistic regression modeling, where a single value is estimated for each coefficient, in Bayesian modeling, coefficients reflect averages calculated over the probability distributions output by each of the many iterations of the model.

The models presented here contain two major departures from the frequentist binomial logistic regression models that sociolinguists are accustomed to. The first stems from their Bayesian nature. Model coefficients are not reported with *p*-values to reflect the probability that the result evident in the data would hold were the null hypothesis true of the wider population. Instead, researchers report 95% Highest Posterior Density (HPD) intervals, or “credible” intervals: intervals in which 95% of the posterior probability density lies. If the 95% credible interval for a predictor does not include 0, we can be reasonably confident that the predictor of interest has a non-zero effect on the data, i.e., the probability of the coefficient for the predictor of interest being non-zero is 0.95. In this way, Bayesian models can be used to estimate the probability of a given parameter taking on a specific value. As Grafmiller et al. (2018) argue, the philosophy behind the Bayesian approach to statistical analysis—estimating the probability of a hypothesis given the data, rather than the probability of one's data given a (null) hypothesis—is intuitively easier to grasp than the traditional frequentist method. The second departure from traditional sociolinguistic modeling is seen in the output of the multinomial model for *has*. Because multinomial logistic regression compares the output of each non-default variant to the default variant, each predictor in a three-way multinomial model has a set of two coefficients, one for each of the non-default variants as compared to the default. Both are interpreted as in binomial logistic regression: again, a positive coefficient favors use of the variant in question over the default; a negative coefficient favors the default over the variant in question. It is possible, for instance, for both non-default predictors to have positive coefficients, indicating that both are favored over the default for a given level of an independent variable.

Running MCMCglmm models requires setting prior probabilities. Following the researchers cited at the beginning of this subsection, I used weakly informative priors defined following the specification for categorical distributions given in Hadfield (2010b, p. 21–24). Another aspect of MCMCglmm that must be set by the user is the number of iterations the model runs for. For the *is* data, I ran 60,000 iterations, sampling every 50th iteration, and discarding the first 10,000 to correct for initial sampling bias (the “burn-in” period). This left 1,000 posterior estimates of each parameter, from which averages and 95% credible intervals are calculated and presented in the following section. For *has*, where there is much less data, I ran 600,000 iterations, sampling every 250th, and discarding the first 50,000. This left 2,200 estimates. Both models were checked for convergence by using strategies to assess autocorrelation suggested in Levshina (2015a, 2016). Checking for autocorrelation (i.e., non-convergence) in each model using the autocorr() function in R as well as by visually examining trace plots of the model's parameters revealed that the model chains had mixed well. Model specifications are provided in the Supplementary Material.

Following Grafmiller et al. (2018), model accuracy was assessed by comparing predicted values generated by the model to observed values for each data point. This allows the construction of a confusion matrix and the calculation of accuracy rates (percent of predicted forms which were correct) and recall rates (percent of observed forms which were correctly predicted). For the binomial model, I also use these predicted values to calculate two measures of model predictive accuracy: Somers' D, which calculates the correlation between the observed values and the log odds of using the default variant for each data point, and the corresponding receiver operating characteristic curve area C.

For both verbs, *contracted* was taken as the default level of the response. If the two-stage analysis of *has*-contraction proposed by MacKenzie (2013) is correct, then we expect to see two choices patterning the same way: the choice between contracted and full forms of *has*, and the choice between contracted and intermediate forms of *has*. This is because the analysis posits a single stage of choice at which speakers decide between using a contracted form and using a full form, which itself may or may not eventually become an intermediate form. Accordingly, the environments in which speakers prefer a contracted form of *has* should equally be the environments in which speakers disprefer the other two forms of *has* (see McLaughlin, 2014 for a very similar approach to the contraction and deletion of *is* in AAVE). Additionally, the models can also answer the question of whether the factors that lead speakers to choose contracted forms of *has* are the same as those that lead speakers to choose contracted forms of *is*, again as suggested by MacKenzie (2013). This would lend further support to the two-stage analysis, opening up the possibility that contraction of *is* and *has* can be unified as a single abstract alternation between contracted and full, with intermediate forms being derivative, stemming from a later process.

To this end, in the next section, I first present the results from the *is* model, and then present the results of the *has* model, to answer the two questions of whether the same environments prefer contracted forms of both verbs, and whether those same environments equally disprefer the two non-contracted forms of *has*.

## 4. Results

**Figure 1:**
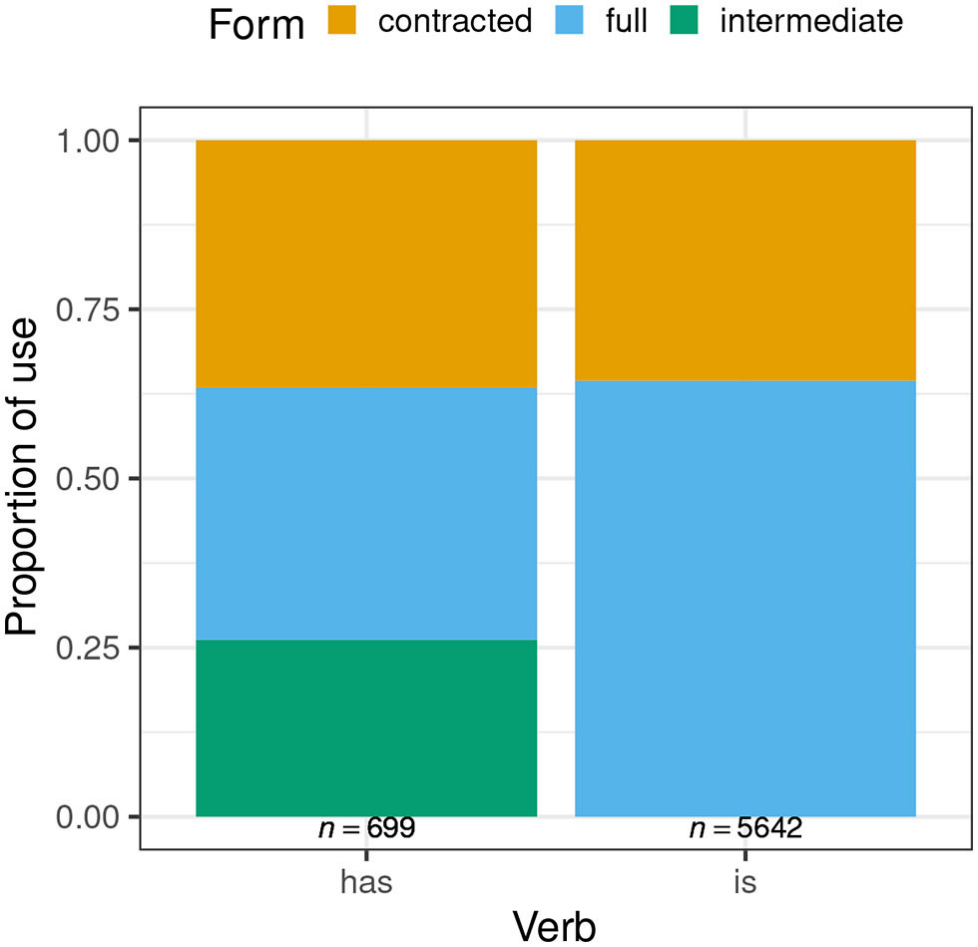
Distribution of variants of post-NP *has* and *is*.

Before turning to the MCMCglmm outputs for each individual verb, it is instructive to consider the overall rates of variant use. Figure 1 shows this. It is immediately apparent that contracted forms of each verb (represented by the orange [uppermost] sections of each bar) are used at an almost identical rate (*has*: 36.6%, *is*: 35.5%). When the two non-contracted forms of *has* are grouped together and opposed to the contracted form, a chi-square test finds no significant difference in distribution of forms between the two verbs (χ^2^ = 0.241, df = 1, *p* = 0.623). This replicates the finding from MacKenzie (2013), but with a considerably larger data set. It also constitutes a first piece of evidence in support of that earlier analysis, under which a first step of choice between contracted and other form(s) applies in a similar way across verbs. Subsequent evidence in favor of—and against—this analysis will be taken from the factors that condition speakers' choice of contracted vs. other forms, to be discussed on a verb-by-verb basis in the two subsections that follow.

### 4.1. *Is*

#### 4.1.1. Model Predictions and Accuracy

The binomial model of *is*-contraction predicts verb form with a high degree of accuracy (C = 0.875, D = 0.749). Table 2 shows the confusion matrix of predicted and observed forms. The model predicts the correct form 80% of the time; this is a significant increase over simply guessing the most frequent form every time, which would give an accuracy rate of 64% (*p*_*binom*_ < 0.001). We can thus be confident that the model is a good fit for the data.

**Table 2:** Confusion matrix for *is*-contraction.

	**Predicted**		
	**Contracted**	**Full**	**Total**
**Observed**			
Contracted	1,288	718	2,006
Full	423	3,213	3,636
Total	1,711	3,931	5,642

#### 4.1.2. Results

**Figure 2:**
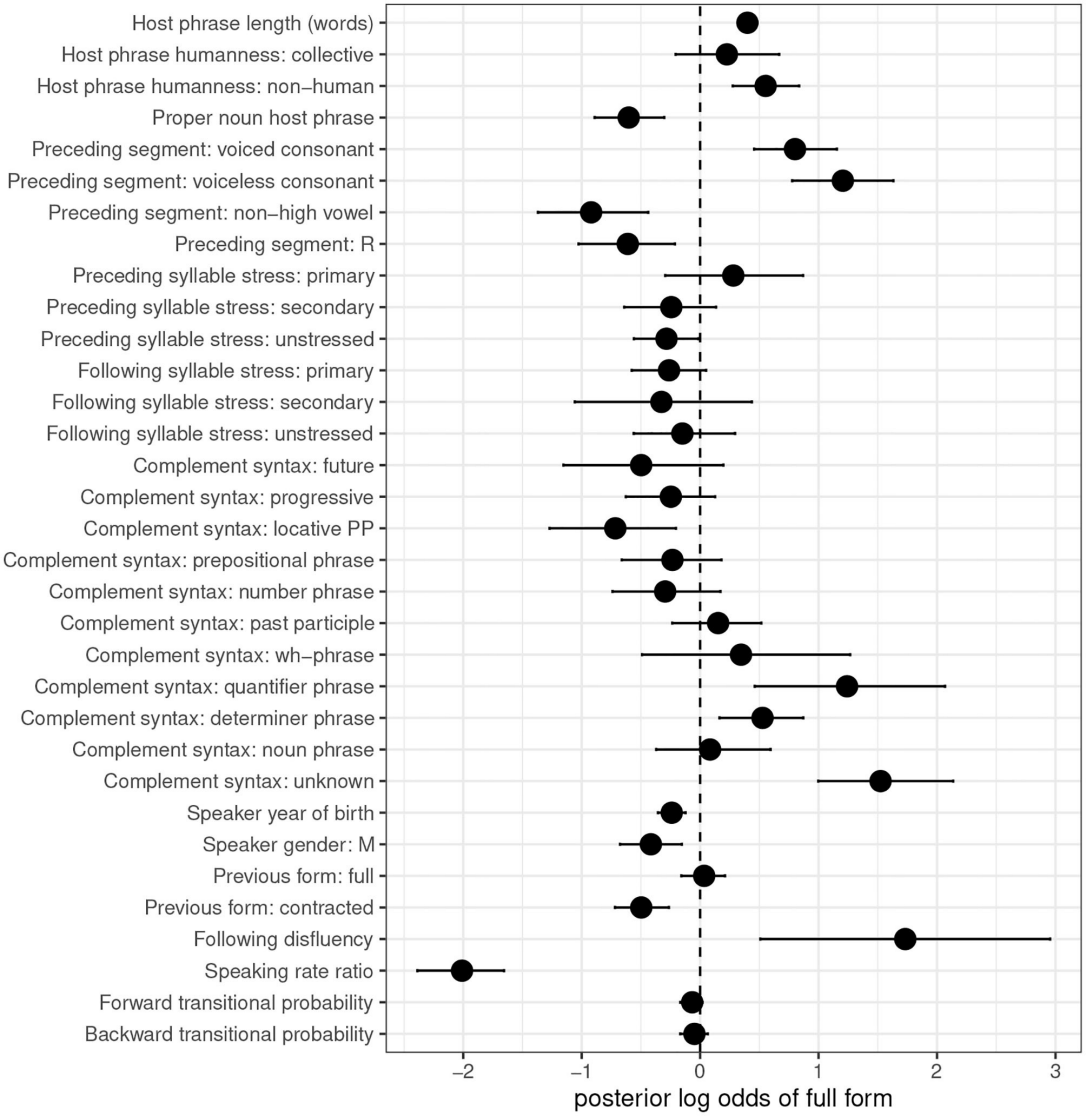
Posterior means and 95% credible intervals for fixed effect predictors, *is*. Default level of dependent variable: contracted form. Points represent posterior log odds of the given predictor on use of the full form.

Figure 2 provides a graphical representation of the estimated log odds of full form usage, along with 95% credible intervals, for each predictor in the *is* model. Table 3 provides the same information—the posterior means and 95% Highest Posterior Density (credible) interval boundaries for each predictor—along with two probabilities, in the last two columns: the probability that the true coefficient for the predictor is less than 0 (i.e., that the predictor favors the use of the default—contracted—form), and the probability that the true coefficient for the predictor is greater than 0 (i.e., that the predictor disfavors use of the contracted form and favors use of the full form). This can help contextualize the results presented visually: even a predictor whose 95% credible intervals cross 0 may nonetheless be predicted with fairly high probability to influence the variation.

**Table 3:** Model estimates for predictors influencing *is*-contraction.

	**Posterior mean**	**l-95% HPDI**	**u-95% HPDI**	**p(β < 0)**	**p(β > 0)**
(Intercept)	1.6290118	0.8140859	2.3619368	0.000	1.000
Host phrase length (words)	0.3990877	0.3373437	0.4623129	0.000	1.000
Host phrase humanness: collective	0.2256141	−0.2071112	0.6676530	0.149	0.851
Host phrase humanness: non-human	0.5528309	0.2753459	0.8361011	0.000	1.000
Proper noun host phrase	−0.6033297	−0.8910730	−0.3031213	1.000	0.000
Preceding segment: voiced consonant	0.8008427	0.4555751	1.1543951	0.000	1.000
Preceding segment: voiceless consonant	1.2047807	0.7769507	1.6319018	0.000	1.000
Preceding segment: non-high vowel	−0.9206464	−1.3695070	−0.4383050	1.000	0.000
Preceding segment: R	−0.6110647	−1.0271768	−0.2129865	0.997	0.003
Preceding syllable stress: primary	0.2800213	−0.2950003	0.8693097	0.164	0.836
Preceding syllable stress: secondary	−0.2439706	−0.6405935	0.1341551	0.892	0.108
Preceding syllable stress: unstressed	−0.2840958	−0.5597557	−0.0136968	0.983	0.017
Following syllable stress: primary	−0.2628040	−0.5776638	0.0503263	0.948	0.052
Following syllable stress: secondary	−0.3271295	−1.0587807	0.4366904	0.798	0.202
Following syllable stress: unstressed	−0.1502357	−0.5612891	0.2949292	0.756	0.244
Complement syntax: future	−0.4980775	−1.1548223	0.1962291	0.915	0.085
Complement syntax: progressive	−0.2476477	−0.6272430	0.1268231	0.906	0.094
Complement syntax: locative PP	−0.7163825	−1.2717282	−0.2043430	0.995	0.005
Complement syntax: prepositional phrase	−0.2341470	−0.6619528	0.1804951	0.850	0.150
Complement syntax: number phrase	−0.2955229	−0.7405061	0.1714253	0.902	0.098
Complement syntax: past participle	0.1516862	−0.2355767	0.5163777	0.213	0.787
Complement syntax: wh-phrase	0.3449906	−0.4907484	1.2669180	0.226	0.774
Complement syntax: quantifier phrase	1.2402996	0.4602654	2.0678252	0.000	1.000
Complement syntax: determiner phrase	0.5265879	0.1631096	0.8713721	0.000	1.000
Complement syntax: noun phrase	0.0834676	−0.3707944	0.5944527	0.373	0.627
Complement syntax: unknown	1.5231752	0.9971601	2.1368757	0.000	1.000
Speaker year of birth	−0.2399602	−0.3561114	−0.1235707	1.000	0.000
Speaker gender: M	−0.4164843	−0.6765843	−0.1543645	0.998	0.002
Previous form: full	0.0339510	−0.1586210	0.2101696	0.379	0.621
Previous form: contracted	−0.4972574	−0.7191570	−0.2636629	1.000	0.000
Following disfluency	1.7319568	0.5081198	2.9550253	0.002	0.998
Speaking rate ratio	−2.0105721	−2.3881814	−1.6560303	1.000	0.000
Forward transitional probability	−0.0673087	−0.1676491	0.0205876	0.914	0.086
Backward transitional probability	−0.0482657	−0.1679330	0.0646831	0.785	0.215

*Default form: contracted. Posterior means are log odds estimates of use of the full form. “l-95% HPDI” and “u-95% HPDI” are lower and upper bounds, respectively, of the 95% credible intervals, the areas in which 95% of the posterior probability density lies. “p(β < 0)” and “p(β> 0)” reflect the posterior probability that the coefficient of a given predictor is negative (favoring the contracted form) or positive (favoring the full form), respectively*.

Four predictors pertaining to the syntax, semantics, and phonology of the host phrase have clear effects on the variation. First, as previous research has found, longer noun phrase subjects disfavor contraction: the positive coefficient for host phrase length in words reflects increased likelihood of full form use with longer subjects. The tight credible intervals make this one of the most reliable predictors in the study.

Second, compared to human host phrases, non-human host phrases favor the use of the full form, replicating McLaughlin's (2014) analysis of a subset of the present data. Though collective host phrases also show a positive coefficient, the 95% credible intervals for this predictor cross 0: collective subjects aren't differentiated from human ones, but non-human subjects are.

Third, contra McLaughlin (2014), we also find an effect of host phrase proper nounhood. Full forms are disfavored (so, contraction is favored) with proper noun host phrases. This is a previously unobserved finding, but, when taken together with the effect of host phrase humanity, it is consistent with typological research, which has found that human and proper noun referents are at the higher end of an animacy scale together (Comrie, 1989, p. 195–196). These two predictors thus provide evidence that animate host phrases promote contraction. It's not immediately clear why an animacy effect should necessarily go in this direction, and most of the research on animacy effects in English concerns word order variation, rather than phonological reduction, so it cannot offer a useful precedent. What is clear, however, is that human and proper noun host phrases affect *is*-contraction in a comparable way, and their robust effects suggest that future work on *is*-contraction must include these predictors for a full account of the variation.

Fourth, all four levels of the preceding segment predictor show coefficients either below or above the 0 line. Preceding consonants favor full forms; preceding non-high vowels and /ɹ/ favor contracted forms. This corroborates Labov (1969), and suggests a pressure on *is*-contraction to maintain CVCV syllable patterning. It also suggests that post-vocalic /ɹ/ in American English behaves as a vowel, at least where contraction is concerned. The results also offer some support for Spencer's (2014) finding that contraction is sensitive to the voicing of a preceding consonant. The coefficient for voiceless consonants is higher than that for voiced consonants, although the credible intervals do overlap. The coefficient for a preceding high vowel, absent from Figure 2 and Table 3, can be calculated to be −0.474 by summing the remaining coefficients and multiplying by −1; this gives us the following hierarchy of preceding segments on conditioning contraction, from most contraction to least:

non-high vowel > /ɹ/ > high vowel > voiced consonant > voiceless consonant

The lower placement on the hierarchy of high vowels compared to non-high vowels corroborates my experience when coding that a preceding high vowel might lead the analyst to be more likely to hear a full form than otherwise. Still, its negative coefficient aligns with the other vocoids in favoring contracted over full forms.

The stress of syllables surrounding the contractable verb plays a minimal role, if any, in conditioning contraction. All credible intervals for surrounding syllable stress levels cross 0, with the exception of unstressed preceding syllables, which display a negative coefficient that just avoids crossing the 0 line (upper bound: −0.014). This suggests a weak favoring effect of a preceding unstressed syllable on *is*-contraction, which is in keeping with Anttila's (2017) proposal that contraction will be more likely when the contractable verb is adjacent to an unstressed syllable. Anttila's (2017) proposal is also (weakly) supported by the positive coefficient of a preceding primary stressed syllable—favoring full over contracted forms—though the credible intervals cross 0 (with an 84% chance that the coefficient is positive). The effect of prosody on *is*-contraction deserves more careful consideration in future work. A first step would be to annotate surrounding syllable stress based on how syllables were actually phrased in production, rather than based on dictionary transcriptions of word stress in isolation.

Complement syntax shows varying effects on *is*-contraction. Consistent with work on *is*-variation in AAVE, determiner phrases (which roughly map onto previous researchers' “noun phrase” category) favor full forms, while locative prepositional phrases favor contracted forms. Quantifier phrases, which are presumably also likely to have been called “noun phrases” in previous work (as they comprise complements such as *a little bit of everything, all these problems*, and *nothing*), pattern with determiner phrases in favoring full forms (though, surprisingly, noun phrases do not). At the same time, some classic contraction-favoring complements in previous work, such as future and progressive forms, show no reliable difference from 0, as do some new distinctions made for the present study, such as non-locative prepositional phrases, number phrases, past participles, and *wh*-phrases. It remains to be determined in future work whether collapsing any of these categories improves model fit. Anttila's (2017) proposal that the effect of complement syntax on *is*-contraction is an artifact of prosodic differences between complement types also deserves careful consideration. For the time being, one last observation worth noting is the strong positive coefficient of what were coded as “unknown” complements. Those cases where the speaker changed direction or restarted their sentence between uttering the verb and its complement strongly favor full forms. This finding has not previously been reported; I return to it later in this subsection.

The two sociodemographic predictors both have negative effects on the use of full forms. Rates of contraction are higher among younger speakers and among speakers identified by the corpus as male. This suggests an age-graded variable (Labov, 2001), though we know little about whether *is*-contraction carries social value, in the way that other age-graded variants are thought to do (Wagner, 2012). This remains an additional area for future research.

Contracted forms show persistence: a speaker who has just uttered a contracted form will be more likely to produce another one, relative to their likelihood of producing a contracted form as their first token of the conversation. There is no comparable persistence effect of full forms, however: production of a full form has no influence on whether a speaker will produce another. This is consistent with other work that has found the less frequent variant of a variable to trigger a stronger persistence effect than the more frequent one (see Tamminga, 2014, p. 97–117 for a review and some additional findings). Contracted forms surface only 36% of the time in the *is* data, suggesting that their persistence may be a surprisal effect.

A following disfluency (*uh* or *um*) strongly favors a speaker's using a full form. This finding is reminiscent of the full form-favoring effect of unknown complements mentioned three paragraphs earlier, and constitutes another previously-undocumented effect on contraction of *is*. One possible interpretation of these two findings is that a speaker's failure to plan the word following the target effectively causes the target to become phrase-final. Phrase-final position is an environment that disallows contraction (King, 1970). That said, there could also be a prosodic explanation: perhaps verbs in these environments bear more stress, disfavoring contraction (Anttila, 2017). The disfavoring effect of upcoming uncertainty on contraction should be probed further in future work; it connects to other research on the effects of production planning on sociolinguistic variation (e.g., Tanner et al., 2017).

This study is the first to find a clear, strong effect of speaking rate on *is*-contraction, with more contraction in faster speech relative to a speaker's average. This could be an effect of prosodic phrasing. There is some evidence that faster speech correlates with longer phrases (e.g., Quené, 2008). And if contraction requires the contractable verb and an adjacent word to be phrased together, as Anttila (2017) proposes, faster speech may make it more likely that speakers phrase their utterances in such a way that effects this. This is yet another indication that the effect of prosodic phrasing on contraction is a rich area for future study.

Finally, both of the measures of transitional probability show credible intervals that cross 0. This means that, in contrast to several previous studies, the Switchboard data show no predictability effects on *is*-contraction. However, a crucial distinction between those studies and this one is the stringent restriction on host phrases used here. I included no token whose host phrase was any sort of pronoun, with “pronoun” defined broadly to include indefinite pronouns. This contrasts, for instance, with Spencer (2014), who also restricted her data to non-pronominal subjects, but included indefinite pronouns among them—and found the expected predictability effects. This suggests that the predictability effects uncovered in previous work may in fact be better attributable to syntactic differences in the types of host phrases that were included in the data. A useful test would be to include tokens with indefinite pronoun hosts among the data used here, and see whether the transitional probability results change.

### 4.2. *Has*

#### 4.2.1. Model Predictions and Accuracy

The goodness-of-fit statistics presented for the *is* model cannot be calculated for a multinomial model, but we can still examine the *has* model's predictive accuracy. Table 4 shows the confusion matrix of predicted and observed forms for *has*. These predictions were obtained by identifying, for each data point, which of the three forms was most probable according to the model.

**Table 4:** Confusion matrix for *has*-contraction.

	**Predicted**			
	**Contracted**	**Full**	**Intermediate**	**Total**
**Observed**				
Contracted	180	45	31	256
Full	52	173	36	261
Intermediate	52	58	72	182
Total	284	276	139	699

The rate of predictive accuracy for *has* is noticeably lower than it was for *is*, presumably a result of the smaller number of tokens and the difficulty imposed on the model of having to make three choices rather than two. The model predicts the correct form only 61% of the time, compared to the *is* model's 80%. Still, this 61% accuracy rate is a significant increase over simply guessing the most frequent form every time, which would give an accuracy rate of 37% (*p*_*binom*_ < 0.001).

Examining the recall rates shown in the rows of Table 4, we can see that the model does a much better job of predicting contracted forms (70% predicted correctly) and full forms (66% predicted correctly) than intermediate forms (only 40% predicted correctly). This may be attributable to the lower rate of representation of intermediate forms in the data (26% of observed forms compared to 37% for both full and contracted). But it also suggests that the predictors included in the present study, while reasonably appropriate for modeling occurrence of contracted and full forms of both verbs under study, may not be the best predictors for modeling occurrence of intermediate forms.

#### 4.2.2. Results

The model of *is*-contraction allowed us to interrogate which factors condition the choice between contracted and full forms of *is*. By contrast, the multivariate model of *has*-contraction allows us to investigate the factors conditioning the choice between contracted and full forms, **and** the factors conditioning the choice between contracted and intermediate forms. However, under the analysis of *has*-variation proposed in MacKenzie (2013) and outlined in section 2.1, these two choices are, underlyingly, a single abstract choice. This means that, if that analysis is correct, the same factors should condition both choices: in other words, the two non-contracted forms of *has* should pattern together. Additionally, if contraction is conditioned in the same way regardless of verb, the same factors that favored *is*-contraction should be at play in *has*-contraction, and those same factors should favor contracted forms of *has* while disfavoring the other two.

**Figure 3:**
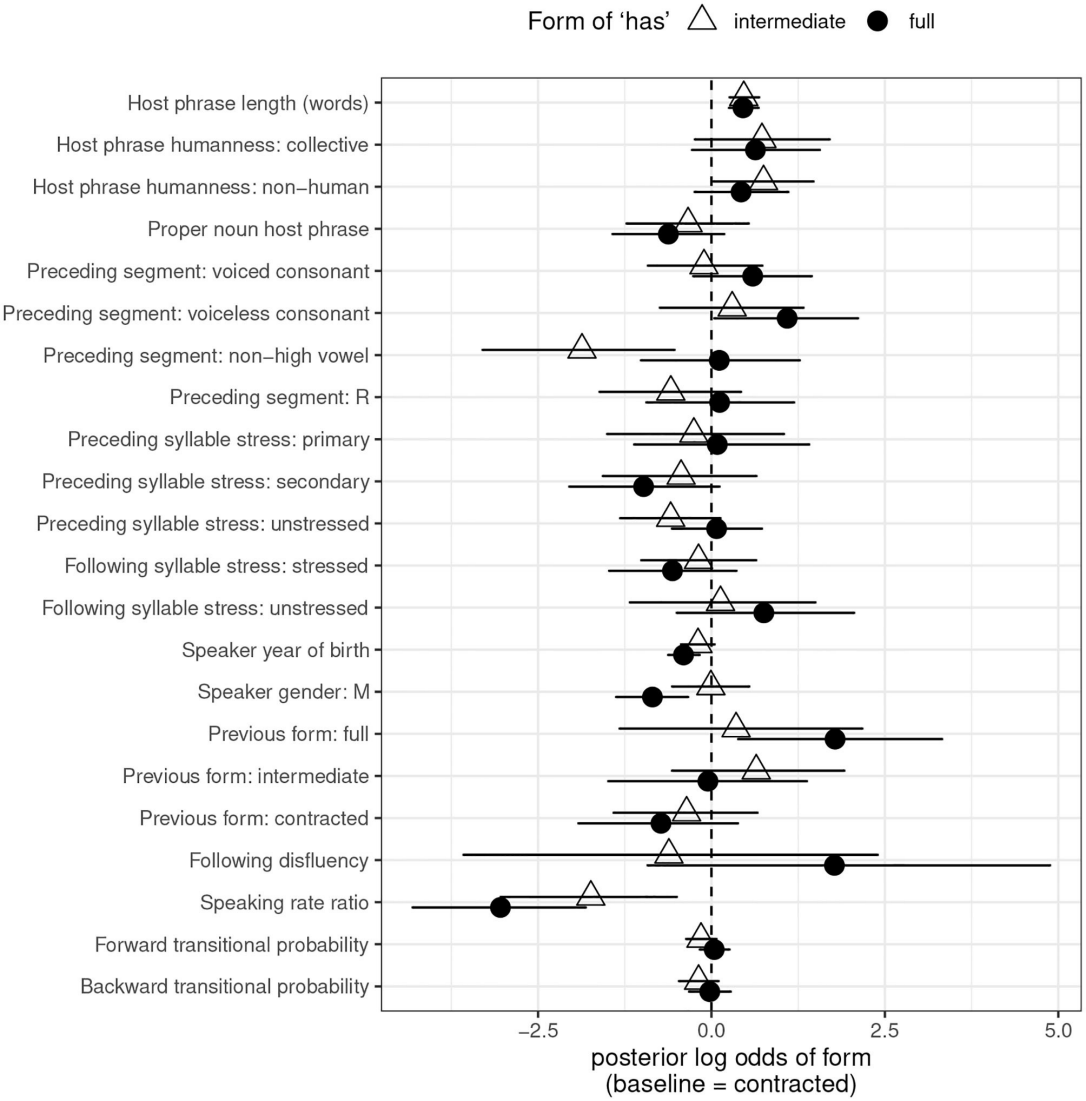
Posterior means and 95% credible intervals for fixed effect predictors, *has*. Default level of dependent variable: contracted form. Points represent posterior log odds of the given predictor on use of the indicated form.

Figure 3 provides a graphical representation of the estimated log odds of full form usage and intermediate form usage, with 95% credible intervals, for each predictor in the *has* model. Because there are two non-default forms to choose from, coefficients and credible intervals are presented for each. Table 5 provides the posterior means, 95% Highest Posterior Density (credible) interval boundaries, and above-/below-0 probabilities for each predictor and each non-default variant. The top half of the table provides the coefficients associated with the choice between contracted and full for each predictor; the bottom half covers the choice between contracted and intermediate. The rows that say simply “Full” and “Intermediate” reflect intercept values: the log odds of using the indicated form over the contracted form when all predictors are set to their default level.

**Table 5:** Model estimates for predictors influencing *has*-contraction.

	**Posterior mean**	**l-95% HPDI**	**u-95% HPDI**	**p(β < 0)**	**p(β > 0)**
Full	1.7562520	−0.4610893	4.1016186	0.0618182	0.9381818
Full - Host phrase length (words)	0.4540251	0.2547957	0.6774660	0.0000000	1.0000000
Full - Host phrase humanness: collective	0.6331986	−0.2787359	1.5609129	0.0840909	0.9159091
Full - Host phrase humanness: non-human	0.4264335	−0.2411717	1.1071036	0.1100000	0.8900000
Full - Proper noun host phrase	−0.6217921	−1.4240903	0.1825051	0.9345455	0.0654545
Full - Preceding segment: voiced consonant	0.5942323	−0.2611305	1.4441186	0.0922727	0.9077273
Full - Preceding segment: voiceless consonant	1.0910646	0.0423456	2.1108090	0.0200000	0.9800000
Full - Preceding segment: non-high vowel	0.1120166	−1.0148022	1.2719679	0.4218182	0.5781818
Full - Preceding segment: R	0.1172536	−0.9374153	1.1907104	0.4131818	0.5868182
Full - Preceding syllable stress: primary	0.0828608	−1.1125612	1.4071405	0.4618182	0.5381818
Full - Preceding syllable stress: secondary	−0.9788212	−2.0480402	0.1140841	0.9627273	0.0372727
Full - Preceding syllable stress: unstressed	0.0735986	−0.5686875	0.7261148	0.4154545	0.5845455
Full - Following syllable stress: stressed	−0.5622588	−1.4733034	0.3584011	0.8863636	0.1136364
Full - Following syllable stress: unstressed	0.7545892	−0.4991466	2.0553168	0.1136364	0.8863636
Full - Speaker year of birth	−0.4034372	−0.6235081	−0.1729058	1.0000000	0.0000000
Full - Speaker gender: M	−0.8515252	−1.3734524	−0.3390654	0.9986364	0.0013636
Full - Previous form: full	1.7816588	0.3862631	3.3237094	0.0072727	0.9927273
Full - Previous form: intermediate	−0.0513835	−1.4860966	1.3737515	0.5186364	0.4813636
Full - Previous form: contracted	−0.7282325	−1.9161779	0.3802393	0.8909091	0.1090909
Full - Following disfluency	1.7721448	−0.9199031	4.8789925	0.1163636	0.8836364
Full - Speaking rate ratio	−3.0423378	−4.3067556	−1.8131940	1.0000000	0.0000000
Full - Forward transitional probability	0.0393089	−0.1678315	0.2581302	0.3640909	0.6359091
Full - Backward transitional probability	−0.0251222	−0.3218068	0.2756846	0.5700000	0.4300000
Intermediate	−0.1687226	−2.4067836	2.2045201	0.5613636	0.4386364
Intermediate - Host phrase length (words)	0.4653107	0.2632404	0.6853951	0.0000000	1.0000000
Intermediate - Host phrase humanness: collective	0.7277026	−0.2379348	1.7027128	0.0686364	0.9313636
Intermediate - Host phrase humanness: non-human	0.7504033	0.0264793	1.4700295	0.0218182	0.9781818
Intermediate - Proper noun host phrase	−0.3374735	−1.2233850	0.5361169	0.7795455	0.2204545
Intermediate - Preceding segment: voiced consonant	−0.1081011	−0.9152518	0.7319900	0.5981818	0.4018182
Intermediate - Preceding segment: voiceless consonant	0.2983763	−0.7416489	1.3259396	0.2890909	0.7109091
Intermediate - Preceding segment: non-high vowel	−1.8667305	−3.2993466	−0.5337918	0.9968182	0.0031818
Intermediate - Preceding segment: R	−0.5862945	−1.6123424	0.4234284	0.8695455	0.1304545
Intermediate - Preceding syllable stress: primary	−0.2542370	−1.5032514	1.0433443	0.6413636	0.3586364
Intermediate - Preceding syllable stress: secondary	−0.4382973	−1.5653899	0.6473658	0.7918182	0.2081818
Intermediate - Preceding syllable stress: unstressed	−0.5886084	−1.3148520	0.1284327	0.9450000	0.0550000
Intermediate - Following syllable stress: stressed	−0.1869565	−1.0106447	0.6422665	0.6736364	0.3263636
Intermediate - Following syllable stress: unstressed	0.1303545	−1.1750509	1.4957722	0.4209091	0.5790909
Intermediate - Speaker year of birth	−0.1942786	−0.4365133	0.0420955	0.9463636	0.0536364
Intermediate - Speaker gender: M	−0.0088502	−0.5666323	0.5440431	0.5104545	0.4895455
Intermediate - Previous form: full	0.3546801	−1.3224632	2.1734082	0.3563636	0.6436364
Intermediate - Previous form: intermediate	0.6451244	−0.5674888	1.9129641	0.1536364	0.8463636
Intermediate - Previous form: contracted	−0.3588215	−1.4097452	0.6613422	0.7500000	0.2500000
Intermediate - Following disfluency	−0.6136116	−3.5729961	2.3940366	0.6559091	0.3440909
Intermediate - Speaking rate ratio	−1.7394001	−3.0362574	−0.5022914	0.9968182	0.0031818
Intermediate - Forward transitional probability	−0.1509607	−0.3625895	0.0697880	0.9172727	0.0827273
Intermediate - Backward transitional probability	−0.1857577	−0.4676284	0.1008418	0.8981818	0.1018182

*Default form: contracted. Posterior means are log odds estimates of use of the indicated form. “l-95% HPDI” and “u-95% HPDI” are lower and upper bounds, respectively, of the 95% credible intervals, the areas in which 95% of the posterior probability density lies. “p(β < 0)” and “p(β> 0)” reflect the posterior probability that the coefficient of a given predictor is negative (favoring the contracted form) or positive (favoring the indicated form), respectively. “Full” and “Intermediate” reflect intercept values*.

A first glance at Figure 3 reveals wide credible intervals for nearly every point. Impressionistically, the credible intervals generally appear wider than those for *is*. This suggests more uncertainty in the *has* model, in keeping with its lower rate of predictive accuracy, and consistent with the smaller data set available for *has* compared to *is*.

Nonetheless, some clear effects are apparent. One of the most notable is the effect of host phrase length. Full and intermediate forms are both favored after longer host phrases, reflecting a disfavoring effect of long host phrases on contracted forms that matches that seen for *is*.

There is somewhat mixed evidence for the predictor of host phrase humanity. As was the case for *is*, both collective and non-human host phrases show positive coefficients on use of the non-default forms. As was also the case for *is*, the credible intervals for collective host phrases cross 0, for both non-default forms, suggesting no reliable difference in contraction rate between human and collective host phrases. But unlike what was the case for *is*, the credible intervals for the effect of non-human host phrases on full forms cross 0. Still, the model does output an 89% chance that the true coefficient for this predictor with this variant is greater than 0, i.e., positive. And the credible intervals for non-human host phrases on intermediate forms do not cross 0 (though they come very close to it, with a lower bound of 0.026). These findings suggest that host phrase animacy could be having the same effect on *has*-contraction as it has on *is*-contraction—that is, disfavoring contracted forms after non-human host phrases—but the results are inconclusive.

We find the same kind of result for proper noun host phrases. As with *is*, the coefficient for both non-default variants is negative. There is a 93% chance that proper noun host phrases favor the contracted variant over the full one, and a 78% chance that they favor the contracted variant over the intermediate one. But again, both credible intervals cross 0.

As with *is*, full forms of *has* are favored over contracted ones after consonants, particularly voiceless ones. (There is a 91% probability that voiced consonants favor full over contracted forms, though the credible interval crosses 0; there is a 98% probability that voiceless consonants favor full over contracted forms, though the credible interval approaches 0, with a lower bound of 0.042). In contrast to *is*, the disfavoring effect of vocoids (vowels and /ɹ/) on full forms of *has* is not in evidence—but preceding vowels do disfavor **intermediate** forms of *has*. All of these findings can be unified if we think of contraction as a phenomenon that seeks to minimize word-final consonant clusters and vowel-vowel hiatus. Consonant-final host words will disfavor contracted forms of any verb, which cliticize to their host word and create word-final consonant clusters. And vowel-final host words will disfavor vowel-initial verb forms which create hiatus: that is, full forms of *is*, and intermediate but not full forms of *has*. These ideas were first proposed by Labov (1969), and continue to find support in this larger, multi-verb data set.

As with *is*, surrounding syllable stress does not play a role in conditioning *has*-contraction. All credible intervals cross zero.

Where full forms are concerned, social factors behave in an identical way between the two verbs. Full forms of *has* are disfavored among younger speakers and by male speakers, as they were for *is*. But intermediate forms of *has* do not follow suit. The credible intervals for both predictors on intermediate forms cross 0, suggesting no influence of these predictors on use of intermediate forms, but a demonstrable influence on speakers' choice between contracted and full.

The persistence effect that can be demonstrated for *has*-contraction takes a different shape than that for *is*-contraction, where a previous contracted form boosted the likelihood of a speaker using a subsequent contracted form. Here, a previous full form boosts the likelihood of a speaker using a subsequent full form. I return to this discrepancy in section 5.

There is an 88% chance that *has* shows the same favoring of full forms in pre-disfluency position as *is*, though with only 14 pre-disfluency tokens in the *has* data, the model understandably shows uncertainty, with very wide confidence intervals. No comparable effect can be demonstrated for intermediate forms of *has*, but again, token counts are very low.

Finally, the last three predictors all accord with the results for *is*. A faster speaking rate relative to a speaker's baseline disfavors both full and intermediate forms, meaning that faster speech favors contracted forms—exactly the effect that was found for *is*. Neither measure of predictability has an effect on either of the non-default variants.

## 5. Discussion

This paper started out with three questions:

1. Which factors condition the alternation between contracted and full forms of *is*?
2. Do those same factors condition the alternation between the contracted form of *has* and its two other possible forms?
3. Does the patterning of *has* lend support to the analysis under which the three surface forms are derived from two stages of binary choice?

Speaking to questions 1 and 2, section 4 finds a number of predictors, some novel and some well-documented, to condition variation in *is*. These include phonological and semantic properties of the verb's host phrase, speaker sociodemographic factors, and characteristics of the speaking situation, such as speech rate and persistence. Most of these also affect variation in *has*, in similar ways. Specifically, of the thirteen predictors examined for both verbs, six of them have non-null effects on both (host phrase length, preceding segment, year of birth, sex/gender, persistence, and speaking rate); a further four have null effects on both (preceding syllable stress, following syllable stress, forward transitional probability, backward transitional probability); and the remaining three show the same patterning for both verbs, but the *has* credible intervals cross the 0 line (host phrase humanness, host phrase proper nounhood, following disfluency).

But the answer to the third question is not as straightforward. To recap, MacKenzie (2013) provided an analysis of intermediate forms of *has* under which they were derived from full forms of *has*. Under this analysis, three forms were derived via two binary choices: first, a choice between contracted and full; second, a choice between full and intermediate. This is a common approach in variationist sociolinguistics to modeling three-way variation (Sankoff and Rousseau, 1989), and MacKenzie (2013) drew on two pieces of evidence to support it in the case of contraction. First, contracted forms are used at near-identical rates for *is* and for *has*. This is consistent with there being a first stage of choice between contracted and any other forms, and with this first stage of choice having an identical rate of application across verbs. As was shown at the beginning of section 4, this holds up in the present data. The second piece of evidence was the patterning of forms by host phrase length. Contracted forms of both verbs were disfavored after longer host phrases, while both full forms and intermediate forms (of *has*) were favored. Again, this holds up in the present data.

Additional support for this analysis in the present study comes from speaking rate, another factor that patterns in the same way: faster speech favors contracted forms of *is* over full forms, and contracted forms of *has* over full and intermediate forms. Weaker evidence in support of the two-stage analysis comes from the predictors of host phrase humanness, host phrase proper nounhood, and year of birth. For all of these, the model coefficients for full and intermediate forms of *has* have the same polarity as each other and as the full form of *is* (either all positive or all negative), but the credible intervals for *has* cross 0 for most of them.

But not all predictors pattern with a contracted vs. full + intermediate split, as the two-stage analysis would predict. In fact, each possible grouping of the three variants of *has* is attested in the data. To discern this, I ran a second multinomial model on the *has* data, with intermediate forms set as the default (reference) level. In this model, coefficients for full forms tell us which factors condition the choice between full and intermediate—the second-stage choice under MacKenzie's (2013) model. The results of this second *has* model are omitted for space reasons, but are available in the Supplementary Material.

This second model reveals that, for some predictors, contracted and intermediate forms pattern together in opposition to full forms. This holds for speaker gender: male speakers favor contracted forms over full **and** intermediate forms over full. This suggests that the gender effect on contraction operates on a distinction between full forms and forms that are phonologically reduced in some way. Even more interesting is the attested persistence effect for *has*. Recall that the persistence effect for *is* showed contracted forms begetting contracted forms, but without a concomitant persistence effect for full forms, which I attributed to contracted forms' being the less commonly used variant of *is*. The persistence effect for *has* is as follows: when a previous form is full, full forms are more likely compared to both contracted and intermediate forms, but neither of the other two variants triggers any persistence effects itself. As with the gender effect, this is interpretable as a full vs. reduced split in variant patterning. And, analogous to what we found for *is*, full forms are in the minority when we split the variants in that way: 37% of forms of *has* are full, compared to 63% which are reduced (i.e., contracted or intermediate). We can unite both verbs' persistence behavior by saying that persistence operates on a full vs. reduced division of variants, and takes the shape of the minority variant in this dichotomy triggering further instances of itself.

Finally, one predictor operates on intermediate as opposed to contracted and full forms. This is preceding segment, specifically, the effect of a preceding vowel. Preceding vowels favor contracted and full forms over intermediate forms of *has*, but play no role in the choice between contracted and full. This again makes sense in light of what was found for *is*, where a preceding vowel disfavored the use of full forms, likely due to a hiatus-avoidance strategy. For *has*, the intermediate form—the only one of the three that is vowel-initial—is disfavored after vowels, but a preceding vowel has no effect on whether a speaker will choose either of the two consonant-initial forms.

These findings complicate the original analysis of *has*-variation put forth by MacKenzie (2013). On the one hand, some predictors do support the proposal that speakers first make a choice between a contracted form of *has* and a full form, which may or may not become intermediate at a later stage of the derivation. On the other hand, effects like the one for a preceding vowel cannot be accommodated. This predictor shapes the choice between contracted and intermediate forms, but under the two-stage analysis, there is no point at which a speaker ever has to decide between using a contracted or an intermediate form. Intermediate forms haven't been derived at the point at which a speaker chooses whether to use a contracted form or not. And yet the findings show us that certain conditioning factors do operate on such decisions.

All of this appears to suggest that variation in *has* is a three-way choice for speakers, between full, intermediate, and contracted forms. But different predictors favor or disfavor different types of forms. Some predictors operate on the distinction between full and phonologically reduced forms: that is, between full on the one hand, and intermediate and contracted on the other. Other predictors are sensitive to whether a form is vowel-initial or not, operating on the distinction between intermediate on the one hand, and full and contracted on the other. And a final set of environments—those that were originally taken to support the two-stage analysis, because they show contracted forms patterning in opposition to full and intermediate ones—can be interpreted as operating on the distinction between non-syllabic and syllabic forms. This last set of environments is perhaps the most interesting one, because the apparent syllabicity effect suggests a prosodic aspect to the variation. And, indeed, the predictors that are sensitive to variant syllabicity include host phrase length and speaking rate, both of which may have their source in prosodic phrasing (Quené, 2008; Anttila, 2017).

As a result, the *has*-contraction findings cast the *is*-contraction findings in a new light. Studying *has* introduces a third form, the intermediate form, which is syllabic (like full), phonologically reduced (like contracted), and vowel-initial (like neither). Observing how it patterns with respect to the other two variants can help us understand which attributes of a form the conditioning factors are sensitive to. For instance, by studying persistence effects on the two verbs, we learn that persistence appears to operate over a phonologically full vs. phonologically reduced dichotomy, with whichever form is in the minority of these two categories triggering subsequent instances of itself. Without the data from *has*, the persistence effects on *is* would be ambiguous between this interpretation and two other interpretations: one in which persistence operates on a vowel-initial vs. consonant-initial dichotomy, and one in which persistence operates on a syllabic vs. non-syllabic dichotomy. Comparing *has*-contraction to *is*-contraction has thus given us deeper insight into how the mechanisms that constrain contraction operate.

## 6. Conclusion

This paper has examined variation in phonological form of two tensed verbs in English, *is* and *has*. Both verbs variably surface in a single-consonant contracted form and a form with all phonological material intact. *Has* differs from *is* in allowing a third form, which is reduced compared to the full form of *has*, making it phonologically near-identical to the full form of *is*. This raises questions about whether the different forms of the two verbs will pattern similarly to one another with respect to a number of internal and external factors. And this, in turn, can inform our analysis of how these different forms are related to one another.

I find a number of similarities in the patterning of the two verbs. These include the overall rate at which the contracted form is used, the constraints that affect the variation, and which form(s) those constraints favor. For both of these verbs, this study has uncovered un(der)documented effects on contraction which deserve further investigation, such as the favoring effect of host phrase animacy on contracted forms, and the potential effect of prosodic phrasing in shaping speakers' choice between syllabic and non-syllabic variants. This latter finding connects with other recent work urging more consideration of the role of prosodic information in conditioning variable processes (Kendall, 2013; Tanner et al., 2017).

But I hope the most lasting contribution of this work will be a methodological one. I approached the ternary variation shown by *has* not by grouping the variants into a particular binary opposition, but by allowing them to vary independently in a Bayesian MCMCglmm. And indeed, by doing this, I uncovered evidence that all three logically possible binary oppositions are evident in the data to some degree. This cannot be captured by modeling *has*-contraction as two binary choices, but rather suggests a three-way choice. Ternary variation like this raises important questions about the nature of the linguistic variable, and complicates the “single-input, single-output” formula so common in traditional variationist sociolinguistic research. It is my hope that more researchers working with non-binary variables will make use of the methods employed here, allowing potential variant groupings to come out of the data rather than imposing groupings on the data themselves.

## Data Availability Statement

The raw data supporting the conclusions of this article will be made available by the authors, without undue reservation.

## Ethics Statement

Ethical review and approval was not required for the study on human participants in accordance with the local legislation and institutional requirements. The patients/participants provided their written informed consent to participate in this study.

## Author Contributions

The author confirms being the sole contributor of this work and has approved it for publication.

## Conflict of Interest

The author declares that the research was conducted in the absence of any commercial or financial relationships that could be construed as a potential conflict of interest.

## Footnotes

^1^The level of stress and reduction of the vowel in the syllabic form also varies, but it is standard practice in the variationist literature on contraction to abstract away from this (Labov, 1969) or to study it as a separate phenomenon, independent of contraction (Spencer, 2014).

^2^Numbers in parentheses are speaker identification numbers from the Switchboard corpus. To facilitate readability, disfluencies and repetitions have been removed from example transcripts, and capitalization and punctuation have been added.

^3^This is typically the verb's subject, but in a *wh*-question (e.g., *How old's your son?*) it may be a different constituent.

^4^Such “neutralization environments” are often omitted in variationist work, but some researchers advocate for their inclusion as long as the modeling can account for their potentially exceptional behavior (e.g., Tanner et al., 2017).

^5^This category included four tokens in which the complement of *is* is quotative *be like*, following Haddican and Zweig's (2012) analysis of quotative *be like* as taking a silent *something* quantifier phrase complement.
