# Supplementary material for: Comparing Constraints on Contraction Using Bayesian Regression Modeling
Source: Front Artif Intell. 2020 Aug 12;3:58. doi: 10.3389/frai.2020.00058 (PMC7861313; doi:10.3389/frai.2020.00058)
Supplement: Supplementary file 1 [file Data_Sheet_1.PDF]

Comparing constraints on contraction using Bayesian regression modeling

Supplementary materials

Laurel MacKenzie

New York University

1 Model specifications

1.1 *Is* model

Multinomial mixed-effects logistic regression was implemented using the `MCMCglmm` package in R. The dependent variable was form of *is*, coded as full ([ɪz] or [əz]) or contracted ([z] or [s]). Priors were specified as follows:

```
k_is <- length(levels(is$aux.form))
I_is <- diag(k_is - 1)
J_is <- matrix(rep(1, (k_is - 1)^2), c(k_is - 1, k_is -
    1))
IJ_is <- (1/k_is) * (I_is + J_is)
prior_is = list(R = list(fix = 1, V = IJ_is, n = k_is -
    1), G = list(G1 = list(V = diag(k_is - 1), n = k_is -
    1), G2 = list(V = diag(k_is - 1), n = k_is - 1),
    G3 = list(V = diag(k_is - 1), n = k_is - 1)))
```

The model specification was as follows:

```
n_is = 60000
b_is = 10000
t_is = 50
```

```
is.model <- MCMCglmm(aux.form ~
    host.phrase.length.orth.wds +
        host.phrase.human +
        host.phrase.proper +
        prec.phon + yob.center +
        sex + prev.aux.form +
        norm.speaking.rate +
        foll.disfluency +
        prec.stress +
        complement.type +
        foll.stress +
        prec.aux.fwd.prob.log.resid +
        aux.foll.bwd.prob.log,
    random = ~speaker.recode +
        prec.word.recode +
        foll.word.recode,
    pr = T, data = is,
    family = "categorical",
    prior = prior_is,
    verbose = TRUE, burnin = b_is,
    nitt = n_is, thin = t_is)
```

1.2 *Has* model

Multinomial mixed-effects logistic regression was implemented using the `MCMCglmm` package in R. The dependent variable was form of *has*, coded as full ([hæz] or [həz]), intermediate ([əz]), or contracted ([z] or [s]). Priors were specified as follows:

```
k_has <- length(levels(has$aux.form))
I_has <- diag(k_has - 1)
J_has <- matrix(rep(1, (k_has - 1)^2), c(k_has - 1,
    k_has - 1))
IJ_has <- (1/k_has) * (I_has + J_has)
prior_has = list(R = list(fix = 1, V = IJ_has, n = k_has -
    1), G = list(G1 = list(V = diag(k_has - 1), n = k_has -
    1), G2 = list(V = diag(k_has - 1), n = k_has -
    1), G3 = list(V = diag(k_has - 1), n = k_has -
    1)))

n_has = 6e+05
b_has = 50000
t_has = 250

has.model <- MCMCglmm(aux.form ~
    -1 + trait + trait:(host.phrase.length.orth.wds +
        host.phrase.human +
        host.phrase.proper +
        yob.center +
        sex + prev.aux.form +
        norm.speaking.rate +
        prec.phon + prec.stress +
        foll.disfluency +
        foll.stress +
        prec.aux.fwd.prob.log.resid +
        aux.foll.bwd.prob.log),
    random = ~us(trait):speaker.recode +
```

```
        us(trait):prec.word.recode +
        us(trait):foll.word.recode,
    rcov = ~us(trait):units,
    pr = T, data = has,
    family = "categorical",
    prior = prior_has,
    verbose = TRUE, burnin = b_has,
    nitt = n_has, thin = t_has)
```

2 Model results, *has*, intermediate form default

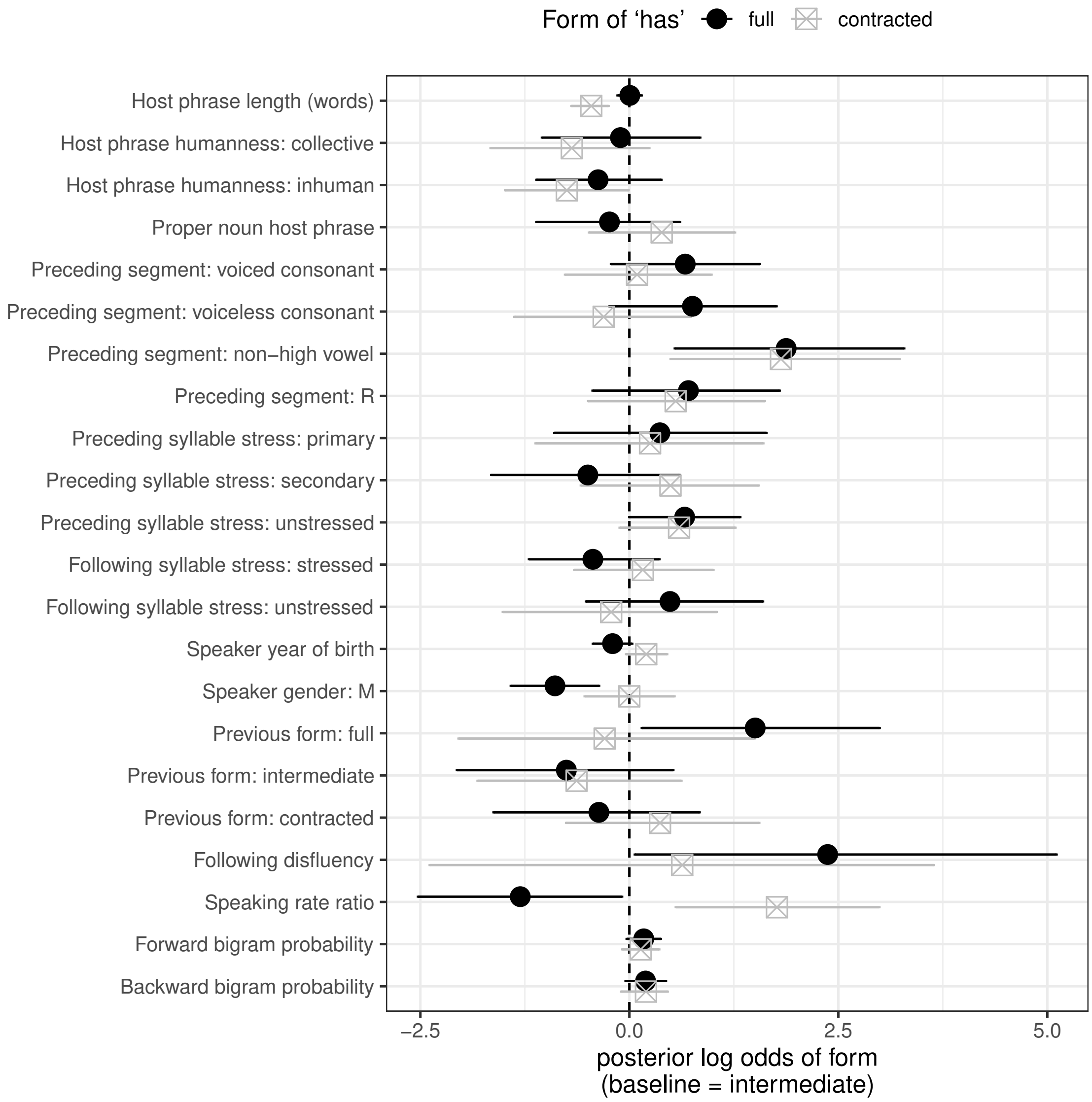

Figure 1: Posterior means and 95% credible intervals for fixed effect predictors, *has*. Default level of dependent variable: intermediate form. Points represent posterior log odds of the given predictor on use of the indicated form. Coefficients for full forms tell us which factors condition the choice between full and intermediate forms, the second-stage choice under MacKenzie's (2013) model. Coefficients for contracted forms redundantly tell us which factors condition the choice between contracted and intermediate forms, something which was seen already in the MCMCglmm of *has* with contracted forms as default. For this reason, the values for contracted forms are given in gray.

Table 1: Model estimates for predictors influencing *has*-contraction. Default form: intermediate. Posterior means are log odds estimates of use of the indicated form. "l-95% HPDI" and "u-95% HPDI" are lower and upper bounds, respectively, of the 95% credible intervals, the areas in which 95% of the posterior probability density lies. "p($\beta < 0$)" and "p($\beta > 0$)" reflect the posterior probability that the coefficient of a given predictor is negative (favoring the contracted form) or positive (favoring the indicated form), respectively. "Full" and "Contracted" reflect intercept values.

	posterior mean	l-95% HPDI	u-95% HPDI	p($\beta < 0$)	p($\beta > 0$)
Full	2.0132879	-0.1531144	4.2812194	0.0354545	0.9645455
Full - Host phrase length (words)	0.0052990	-0.1413992	0.1475939	0.4709091	0.5290909
Full - Host phrase humanness: collective	-0.1066251	-1.0460594	0.8479936	0.5913636	0.4086364
Full - Host phrase humanness: inhuman	-0.3739908	-1.1119579	0.3831002	0.8304545	0.1695455
Full - Proper noun host phrase	-0.2383206	-1.1129903	0.6070984	0.7131818	0.2868182
Full - Preceding segment: voiced consonant	0.6680746	-0.2199797	1.5593622	0.0627273	0.9372727
Full - Preceding segment: voiceless consonant	0.7547575	-0.2426977	1.7610802	0.0709091	0.9290909
Full - Preceding segment: non-high vowel	1.8756387	0.5434705	3.2878290	0.0031818	0.9968182
Full - Preceding segment: R	0.7067527	-0.4403113	1.7986314	0.0959091	0.9040909
Full - Preceding syllable stress: primary	0.3651971	-0.8982520	1.6403198	0.2790909	0.7209091
Full - Preceding syllable stress: secondary	-0.4969074	-1.6526238	0.6006924	0.8204545	0.1795455
Full - Preceding syllable stress: unstressed	0.6613029	-0.0011307	1.3267781	0.0259091	0.9740909
Full - Following syllable stress: stressed	-0.4367475	-1.2013350	0.3593944	0.8645455	0.1354545
Full - Following syllable stress: unstressed	0.4860320	-0.5182797	1.5990718	0.1809091	0.8190909
Full - Speaker year of birth	-0.2010322	-0.4368910	0.0342114	0.9554545	0.0445455
Full - Speaker gender: M	-0.8899526	-1.4182907	-0.3621397	0.9995455	0.0004545
Full - Previous form: full	1.5053699	0.1488758	2.9941335	0.0104545	0.9895455
Full - Previous form: intermediate	-0.7536580	-2.0634088	0.5262179	0.8781818	0.1218182
Full - Previous form: contracted	-0.3646253	-1.6251338	0.8405266	0.7168182	0.2831818
Full - Following disfluency	2.3720411	0.0648183	5.1107390	0.0218182	0.9781818
Full - Speaking rate ratio	-1.3051774	-2.5288665	-0.0866013	0.9809091	0.0190909
Full - Forward bigram probability	0.1721996	-0.0322692	0.3767667	0.0504545	0.9495455
Full - Backward bigram probability	0.1936789	-0.0454668	0.4381145	0.0554545	0.9445455
Contracted	0.0414872	-2.2081830	2.3830004	0.4881818	0.5118182
Contracted - Host phrase length (words)	-0.4563064	-0.6929704	-0.2487713	1.0000000	0.0000000
Contracted - Host phrase humanness: collective	-0.6886141	-1.6605471	0.2396210	0.9277273	0.0722727
Contracted - Host phrase humanness: inhuman	-0.7481549	-1.4864601	-0.0088593	0.9750000	0.0250000
Contracted - Proper noun host phrase	0.3867815	-0.4830539	1.2641347	0.1954545	0.8045455
Contracted - Preceding segment: voiced consonant	0.0921801	-0.7695821	0.9831111	0.4268182	0.5731818
Contracted - Preceding segment: voiceless consonant	-0.3072119	-1.3751170	0.7318176	0.7263636	0.2736364
Contracted - Preceding segment: non-high vowel	1.8134827	0.4932856	3.2314939	0.0018182	0.9981818
Contracted - Preceding segment: R	0.5538143	-0.4928426	1.6202901	0.1531818	0.8468182
Contracted - Preceding syllable stress: primary	0.2475240	-1.1225373	1.6029478	0.3509091	0.6490909
Contracted - Preceding syllable stress: secondary	0.4917725	-0.5809863	1.5468368	0.1859091	0.8140909
Contracted - Preceding syllable stress: unstressed	0.5945201	-0.1164283	1.2690548	0.0468182	0.9531818
Contracted - Following syllable stress: stressed	0.1615993	-0.6601901	1.0061538	0.3522727	0.6477273
Contracted - Following syllable stress: unstressed	-0.2177317	-1.5139809	1.0455879	0.6322727	0.3677273
Contracted - Speaker year of birth	0.2016352	-0.0389991	0.4523611	0.0504545	0.9495455
Contracted - Speaker gender: M	-0.0009599	-0.5347075	0.5405905	0.5031818	0.4968182
Contracted - Previous form: full	-0.2955724	-2.0441717	1.4966211	0.6304545	0.3695455
Contracted - Previous form: intermediate	-0.6313621	-1.8160451	0.6230116	0.8404545	0.1595455
Contracted - Previous form: contracted	0.3665438	-0.7565492	1.5540547	0.2609091	0.7390909
Contracted - Following disfluency	0.6322886	-2.3887052	3.6419787	0.3354545	0.6645455
Contracted - Speaking rate ratio	1.7653114	0.5551297	2.9916656	0.0040909	0.9959091
Contracted - Forward bigram probability	0.1360393	-0.0840107	0.3579107	0.1090909	0.8909091
Contracted - Backward bigram probability	0.1984457	-0.0969646	0.4590367	0.0868182	0.9131818
